# Board task performance: An exploration of micro- and macro-level determinants of board effectiveness

**DOI:** 10.1002/job.743

**Published:** 2011-01-04

**Authors:** Alessandro Minichilli, Alessandro Zattoni, Sabina Nielsen, Morten Huse

**Affiliations:** 1 Department of Management & Technology, Bocconi University, Milan, Italy; 2 Management Department, Parthenope University, Naples, Italy; 3 SDA Bocconi School of Management, Strategic and Entrepreneurial Management Department, Milan, Italy; 4 Department of International Economics and Management, Copenhagen Business School, Denmark; 5 Norwegian School of Management BI, Department of Innovation and Economic Organization, Oslo, Norway; 6 Tor Vergata University, Rome, Italy

## Abstract

This paper addresses recent calls to narrow the micro–macro gap in management research (Bamberger, 2008), by incorporating a macro-level context variable (country) in exploring micro-level determinants of board effectiveness. Following the integrated model proposed by Forbes and Milliken (1999), we identify three board processes as micro-level determinants of board effectiveness. Specifically, we focus on effort norms, cognitive conflicts and the use of knowledge and skills as determinants of board control and advisory task performance. Further, we consider how two different institutional settings influence board tasks, and how the context moderates the relationship between processes and tasks. Our hypotheses are tested on a survey-based dataset of 535 medium-sized and large industrial firms in Italy and Norway, which are considered to substantially differ along legal and cultural dimensions. The findings show that: (i) Board processes have a larger potential than demographic variables to explain board task performance; (ii) board task performance differs significantly between boards operating in different contexts; and (iii) national context moderates the relationships between board processes and board task performance. Copyright © 2010 John Wiley & Sons, Ltd.

## Introduction

Most previous governance studies have been criticized for either adopting under- or over-contextualized views. On the one hand, literature on corporate governance and boards of directors has primarily applied agency theory to explore the impact of board demographics on firm financial performance (Aguilera, Filatotchev, & Jackson, 2008; Daily, Dalton, & Cannella, 2003). The reliance on the agency paradigm, characterized by an extensive focus on board demographics, diverted past research from considering the influence of board processes on board effectiveness (Forbes & Milliken, 1999; Hambrick, v. Werder, & Zajac, 2008; Huse, 2007), and hardly allowed scholars to consider the impact of macro-social contexts on governance practices and behaviors (Aguilera et al., 2008; Aguilera & Jackson, 2003; Lubatkin, 2007). On the other hand, comparative studies in the financial economics and law research traditions relied on arguments based on institutional theory to explore governance mechanisms in different settings; thereby underestimating the importance of organizational contexts in determining the effectiveness of central governance mechanisms such as corporate boards (Lubatkin, 2007).

Following recent calls to narrow the micro–macro gap in management research (e.g., Bamberger, 2008), this paper argues for the co-existence of micro- and macro-level determinants in understanding board effectiveness within corporate governance structures. As such, the study follows the call to go beyond the simple acknowledgment of surrounding phenomena as “error variance” (Bamberger, 2008: 840) and incorporates context- or macro-level determinants in exploring board effectiveness.

Specifically, our purpose is to investigate board processes as micro-level determinants of board effectiveness (Gomez-Mejia & Wiseman, 2007; Zahra, 2007). We measure board effectiveness as board task performance in control and advice, and relate these to three antecedents of board task performance; effort norms, cognitive conflicts and the use of knowledge and skills (Forbes & Milliken, 1999). These constructs are solidly grounded in traditional organizational research (Hackman & Morris, 1975), but find renewed vigor in explaining the task performance of boards of directors, which differ in their characteristics from other decision-making workgroups.

Additionally, we adopt a cross-national or cross-cultural lens (Gomez-Mejia & Wiseman, 2007) to complement existing board research by introducing macro-level determinants of board effectiveness. According to the institutional perspective, human and social behaviors are driven by the influence of country-level institutions, such as norms, routines and historical patterns, which determine isomorphism among individuals and organizations (DiMaggio & Powell, 1983; Scott, 2001). Building on this logic, we investigate the impact of national contexts on board tasks, as well as how context moderates the relationship between processes and tasks.We test our hypotheses on a dataset from a cross-country survey of medium and large Norwegian and Italian firms of comparable sizes across countries. We selected countries that reflect the Scandinavian and French civil law traditions, respectively, because these two contexts differ from each other along several important dimensions, such as shareholders' and creditors' protection (e.g., La Porta, Lopez-de-Silanes, Shleifer, & Vishny, 1998; Lubatkin, Lane, Collin, & Very, 2005), as well as cross-cultural aspects (e.g., Hofstede, Van Deusen, Mueller, & Charles, 2002; Polley, 1988; Waldman et al., 2006). Hence, we follow a long tradition of the cross-national, cross-cultural organizational behavior research, which has considered national contexts as proxies for cultures and institutions (Tsui, Nifadkar, & Ou, 2007).

This paper contributes to existing literature in three related ways. First, we investigate board processes as determinants of board effectiveness in conducting its tasks (Forbes & Milliken, 1999; Huse, 2007; Hambrick et al., 2008) through a large-scale cross-national survey. This approach helps opening the ‘black box’ of corporate boards (Zona & Zattoni, 2007) by avoiding exclusive reliance on secondary data in board research (Daily et al., 2003; Hambrick et al., 2008). Second, following calls to determine “how institutions shape corporate governance” (Aguilera & Jackson, 2003: 449), we offer insights to comparative institutional analysis. Focusing on board processes allows us to go beyond the recognition of cross-national differences among board structures (Aguilera & Cuervo-Cazurra, 2004). Specifically, we investigate how boards engage in their tasks in different contexts, providing evidence for the actual influence of dominant institutional norms and values on board internal mechanisms (Hambrick et al., 2008). Finally, we contribute to the transitioning from a-contextual research (Bamberger, 2008) to the explicit consideration of contexts within the domain of boards and governance research. As such, the study offers novel inputs to the ongoing debate on the ‘embedded model of governance’ (Lubatkin, Lane, Collin, & Very, 2007), which argues for the existence of a close interplay of firm- and institutional- levels in defining governance mechanisms (Aguilera & Jackson, 2003; Lubatkin, 2007; Lubatkin et al., 2007; Scharpf, 1999; Williamson, 2000).

The paper proceeds as follows. We first introduce the organizational-level relationship between board processes and board task performance. Next, we discuss the macro-level institutional settings governing board behavior, leading to our hypotheses on the moderating effects of country contexts on the above relationship. After presenting our methods and results, the paper concludes with a discussion of its contribution to the extant literature and suggestions for future research.

## Theoretical Background

### Board task performance

We follow the tradition of boards and governance research by considering board effectiveness in relation to board task performance (Forbes & Milliken, 1999; Johnson, Daily, & Ellstrand, 1996; Stiles & Taylor, 2001; Zahra & Pearce, 1989). According to this tradition, boards are expected to perform control and advisory tasks. The control tasks follow predictions of agency theory (Fama, 1980; Jensen & Meckling, 1976), suggesting that the primary responsibility of corporate boards is to safeguard shareholders from management misappropriation (Shleifer & Vishny, 1997). From this perspective, boards are groups of independent people with the duty to actively control top executives and the incentives to operate in the interests of firm's shareholders (Fama & Jensen, 1983). To accomplish control tasks, board members should scrutinize top executives' behaviors and actively monitor firm performance to satisfy both shareholders' and stakeholders' expectations (Hillman & Dalziel, 2003). The attention to board control tasks is regaining interest in light of the current global financial crisis. As such, board control is increasingly considered a primary measure of boards' effectiveness, and is thus subject to severe public scrutiny.

At the same time, from a resource dependence perspective (Pfeffer & Salancik, 1978), board advisory tasks relate to the ability of board members to bring additional resources to the firm (Hillman & Dalziel, 2003). According to this view, corporate boards are groups of competent people contributing to boardroom debate by bringing in their experiences, competences and pluralistic perspectives (Forbes & Milliken, 1999). In this respect, board members contribute to strategic decision-making by providing valuable advice and counsel to firm top executives (Daily & Dalton, 1994; Hillman, Cannella, & Paetzold, 2000; Judge & Zeithaml, 1992). The advisory tasks are conceptually rooted in a collaborative model of board of directors, complementing the independent model according to which boards have the primary responsibility to control managers on behalf of shareholders (Westphal, 1999).

### Board processes and board task performance

The understanding of board control and advisory task performance requires identifying predictors of board effectiveness. To this end, we follow calls to explore board behaviors beyond the reliance of demographics as proxies of actual processes (Daily et al., 2003; Forbes & Milliken, 1999; Pettigrew, 1992). The need to explore processes in boards and governance research has been recently advocated by Hambrick et al. (2008), who argue that “behavioral processes” represent the main determinants of governance at the micro-level of analysis. Accordingly, we build on the integrated model of board effectiveness proposed by Forbes and Milliken (1999), which identifies three main board processes as predictors of board task performance: Effort norms, cognitive conflicts and use of knowledge and skills. Grounded in organizational theory (Hackman & Morris, 1975), these processes have mainly been tested in different decision-making workgroups (see Bettenhausen, 1991; Cohen & Bailey, 1997 for a review). Notwithstanding, they are thought to have a peculiar meaning within the context of corporate boards, characterized as “large, elite, episodic decision making groups that face complex tasks pertaining to strategic issue processing” (Forbes & Milliken, 1999: 492). Several aspects characterize boards as peculiar decision-making groups. Specifically, boards are: (a) Larger than other workgroups; (b) made up of a majority of “outsiders”, with primary affiliation in other companies (and thus with a more limited knowledge of company issues); (c) episodic, since they meet from 6 to a maximum of 12 times per year and (d) without a concrete and “tangible” outcome, i.e., boards are not responsible for implementing decisions, and their outcome is entirely “cognitive” in nature. These characteristics make boards particularly vulnerable to “process losses” that prevent them from achieving their full potential (Forbes & Milliken, 1999; Steiner, 1972).

*Effort norms* is a “group-level construct that refers to the group's shared beliefs regarding the level of effort each individual is expected to put toward a task” (Forbes & Milliken, 1999: 493). Recent reviews emphasize the importance of the effort board members devote to preparation, analysis and participation in boardroom debates (e.g., Hambrick et al., 2008). Most board members are busy professionals who face competing demands for their time (Lorsch & MacIver, 1989). As such, the effort devoted to different tasks may vary considerably across boards, with the potential result that board members often act as a “rubber stamp” for managerial proposals (Lorsch & MacIver, 1989; Mace, 1971; Stiles & Taylor, 2001).

As board members often fail to do their “homework”, i.e., do not analyze documents and information provided before meetings (Forbes & Milliken, 1999), the probability of them merely acting as a passive audience in corporate boardrooms increase. The lack of effort is especially relevant in the board context, since boards are decision-making groups composed mostly of outsiders who bring substantial independence at the price of lower inside knowledge of the firm and its strategies (Mallette & Fowler, 1992). Hence, preparation for and participation in board meetings—in terms of carefully scrutinizing information provided by management before meetings, finding autonomously own information regarding issues relevant to the company and actively partaking during meetings with questions—can influence the board's ability to effectively perform its tasks (Forbes & Milliken, 1999; Wageman, 1995). Such efforts ensure constructive and fruitful discussions, thereby improving the quality of decision-making and contributing to the performance of cognitive and intellective tasks (Watson & Michaelsen, 1988). Based on arguments above, we contend that effort norms facilitate both control and advisory tasks. Finding their own information together with careful scrutiny of management reports favor board oversights, whilst in-depth preparation together with active participation during meetings facilitate board advice to management. Thus, we hypothesize the following:

*Hypothesis 1*: Effort norms will have a positive impact on board control and advisory task performance.

*Cognitive conflicts* refer to task-oriented differences in judgment among group members, often manifested in “disagreements about the content of the tasks being performed, including differences in viewpoints, ideas and opinions” (Jehn, 1995: 258). As a result of the effects of conflicts on group effectiveness still being equivocal (De Dreu & Weingart, 2003), Jehn (1995, 1997) proposed an alternative perspective by differentiating between task and relationship conflicts. She noticed that, although relationship conflicts generally decrease satisfaction among group members and negatively interfere with task performance, *task conflicts* can be beneficial to task performance when the group is working on non-routine tasks (Jehn, 1995). The focus on boards of directors as decision-making groups emphasizes the difference between task and relationship conflicts. As groups of highly qualified individuals without hierarchical relationships and meeting only episodically, corporate boards represent a context in which *relationship conflicts* and personal antagonisms are less likely to take place than in other organizational teams. Rather, the non-routiness of tasks performed by corporate boards, along with the complexity of their decision-making process as well as the interdependence among board members, emphasizes the positive effects of task-related cognitive conflicts (Forbes & Milliken, 1999).

Cognitive conflicts can improve decision-making because they facilitate the exchange of information among board members (Amason & Sapienza, 1997). The presence of cognitive conflicts can increase the quality of debate, forcing board members to consider a broader range of alternatives (Forbes & Milliken, 1999). Moreover, cognitive conflicts increase the group members' tendency to scrutinize task issues and to engage in deliberate processing of task-relevant information (De Dreu & Weingart, 2003). This, in turn, may lead to the consideration of additional alternatives, as well as more careful evaluation of these (Eisenhardt et al., 1997). As such, the presence of disagreements and critical investigation among board members can force top managers to justify their strategic proposals and to consider alternative perspectives.

In other terms, while we recognize that conflicts can be either beneficial or detrimental to group performance depending on group characteristics (De Dreu & Weingart, 2003), we also argue that non-routiness of tasks performed in boardrooms emphasizes the positive sides of conflicts among directors. Based on arguments suggesting that cognitive conflicts may lead to better decision-making processes, we hypothesize the following:

*Hypothesis 2*: Cognitive conflicts will have a positive impact on board control and advisory task performance.

*Use of knowledge and skills* refers to “the board's ability to tap the knowledge and skills available to it and then apply them to its tasks” (Forbes & Milliken, 1999: 495). The use of knowledge and skills is associated with the process by which board members' contributions are coordinated, and specifically refers to the flows of information among board members, the clear division of tasks and responsibilities, and the awareness board members should have of each others' competences and areas of expertise (Forbes & Milliken, 1999). This construct differs from the *presence* of knowledge and skills, which conversely refers to the presence of professional (e.g., finance, marketing, accounting, law) and/or firm specific knowledge and expertise. Boards are usually populated by highly competent and reputed individuals; however, the mere presence of knowledge, we argue, does not *per se* mean that board members will use their knowledge (Forbes & Milliken, 1999; Zona & Zattoni, 2007). Rather, effective boards require active use and integration of board members' expertise and skills to enhance group decisions. The collective use of knowledge and skills is particularly relevant when groups are highly interdependent, and when the group shares a sense of collective responsibility for performance outcomes (Wageman, 1995). It gains additional relevance when interdependent groups are also episodic, since the use of knowledge may prevent “process losses” and help board members build on each others' professionalism. Corporate boards represent such groups as board members “must elicit and respect each others” expertise, build upon each others' contributions, and seek to combine their insights in creative, synergistic ways' (Forbes & Milliken, 1999: 496). Hence, the proper use of knowledge and skills is believed to enhance task performance in both control and advice:

*Hypothesis 3*: The use of knowledge and skills will have a positive impact on board control and advisory task performance.

Board effectiveness in control and advisory task performance is also relevant in relation to firm-level outcomes. As discussed above, both control and advice tasks of boards are believed to influence performance by preventing management misappropriation (Shleifer & Vishny, 1997) and bringing qualified advice and counsel to firm top executives (Daily & Dalton, 1994; Judge & Zeithaml, 1992). In doing so, effective boards of directors avoid “distraction” of value by managerial expropriation (through effective control) and, at the same time, allow for maximization of value creation (through effective advice). Hence, we contend that board tasks, as the sole board-level outcome to directly influence corporate financial results (Forbes & Milliken, 1999), are important predictors of firm financial outcomes (Zahra and Pearce, 1989):

*Hypothesis 4*: Board control and advisory task performance will have a positive impact on firm financial performance.

### Governance systems and institutional settings

Governance systems are embedded in national institutional environments (Aguilera et al., 2008; Aguilera & Jackson, 2003; Buck & Shahrim, 2005). Despite law harmonization, financial market integration, and diffusion of codes of best practice, are pushing corporate governance practices to become increasingly similar around the world, they continue to formally and substantially differ across contexts (e.g., Aguilera & Cuervo-Cazurra, 2004; Aguilera & Jackson, 2003; Roe, 2003). Hence, at the macro-level, national contexts potentially exert a significant influence on board task performance and internal processes.

Following institutional arguments, sources of contextual differences are formal institutions and constraints (North, 1990; Whitley, 1992) as well as informal or background institutions (DiMaggio & Powell, 1983). Among the formal institutions, legal regimes are rooted in the legal traditions of nations (La Porta et al., 1998). From a legalistic perspective, Scandinavian (Norway, Sweden, Denmark, and Finland) and French (e.g., France, Spain, Italy, etc.) legal traditions have been shown to differ along several dimensions (La Porta et al., 1998). For instance, national context has been shown to significantly influence the financial systems and consequently the local patterns of corporate ownership (Pedersen & Thomsen, 1997; Thomsen & Pedersen, 1996, 2000; Whitley, 1992).

As for background institutions, different contexts are characterized by specific national cultures. Culture has been defined as ‘a shared meaning system […], during a specific historic period, and in a definable geographic region’ (Triandis, 2000: 146). Culture refers to the “complex of meanings, symbols, and assumptions about what is good or bad, legitimate or illegitimate that underlie the prevailing practices and norms in a society” (Licht et al., 2005: 233). As such, national culture has the potential to influence and shape business practices and styles, and work-related values and behaviors. Based on their national origin, for instance, managers “not only contribute to the collective formulation of cultural norms and views, they experience social reinforcement pressures which bring their individual-level assumptions and preferences into close alignment with those of their native culture” (Geletkanycz, 1997: 617).

### Board task performance and processes in different institutional contexts

From a legal perspective, board task performance is dependent on requirements by corporate laws and voluntary codes of good governance based on a “comply or explain” principle. Thus, board task performance may vary in different legal regimes. In this regard, the Scandinavian system is characterized by higher legal protection and efficiency of the judiciary system compared to the Latin system (La Porta et al., 1998). Hence, in Scandinavian countries corporate boards are likely to feel greater pressures on task performance, especially in control; such pressures stem from both disclosure requirements and legal responsibilities that board members must adhere to.

Besides legal constraints, board task performance may be influenced by cultural traits characterizing different national contexts. Comparative studies on cross national differences in cultures show strong dissimilarities among work-related individual values and behaviors as a consequence of the broader political, sociological and psychological influence of nationality (e.g., Hofstede, 1983, 1984, 2001; Hofstede et al., 2002; see also Egri and Ralston, 2004; Kirkman, Chen, Farh, Chen, & Lowe, 2009; Man and Lam, 2003). For instance, in a study of the perceived goals of business leaders within organizations, Hofstede et al. (2002) show how Latin leaders emphasize family interests, personal wealth and power, while the northern European model of business is driven by responsibility toward employees and society at large. Consistent, we argue that the consideration of stakeholders' interests in Latin countries is relatively lower compared to Scandinavia.

Thus, Scandinavian boards are more involved in both control and advisory tasks. Higher performance in control tasks is grounded in the higher institutional pressures Scandinavian board members experience in terms of disclosure and enforcement of their legal responsibilities. Higher performance in advisory tasks is predicated by a business culture in Scandinavia which traditionally emphasizes an active role of boards within firms (Huse, 1990). Hence, we hypothesize the following:

*Hypothesis 5*: Board performance in both control and advisory tasks will be higher in Scandinavian countries compared to Latin countries.

The impact of context is not limited to board task performance. Isomorphic pressures related to inertial rules and routines embodied in institutional environments (McNulty & Pettigrew, 1999; Ocasio, 1999) and in different national cultures (Hofstede, 2001) also have the potential to significantly influence board processes (Hambrick et al., 2008). In this respect, intriguing insights emerge from the comparison of cultural traits among Scandinavian and Latin countries. In his studies of cultural relativity and its impact on managerial practices and behaviors, Hofstede (1983, 1984, 2001) shows how Latin countries are more individualistic societies, dominated by larger “power distance” and stronger hierarchies in workplaces than Scandinavian countries (Hofstede, 1984). Power distance is related to the “degree of centralization of authority and the degree of autocratic leadership” (Hofstede, 1984: 81), and together with individualism defines the leadership style which is idiosyncratic to a specific national context. In countries characterized by high levels of individualism and power distance (i.e., Latin countries), individual subordinates usually refrain from participating in decision-making. As such, it is expected that leaders will lead “autocratically” (Hofstede, 1984).

In this study, we show how culture may moderate the relationships between board processes and board task performance. In particular, we argue that board processes will be stronger predictors of board effectiveness in individualistic rather than in collectivistic societies. Individualism, for instance, may induce overconfidence in executives' ability to lead the firm, and, as a result, reinforces the importance of individual decision-making over group consensus (Geletkanycz, 1997). Individualism may also parallel opportunism in that it allows room for opportunistic behaviours, since individualistic societies are likely to be characterized by lesser social control than collectivistic ones. In individualistic contexts, the presence of competent and skilful professionals in corporate boardrooms does not ensure the effective use of knowledge. In such contexts, the board leader (CEO and/or Chair) may prefer to assign individual responsibility to him- or herself, rather than eliciting collective use of knowledge and skills. Provided the importance of collective use of such knowledge in highly interdependent groups (Wageman, 1995), the actual active use of knowledge and skills will have a stronger impact on board task performance in individualistic rather than in collectivistic societies.

The opportunistic risks associated with individualism provide further arguments for the higher relevance of effort norms in leading board members' behaviours and force them to fulfil their responsibilities as board members. Hence, boardroom cultures characterized by emphasis on board members' preparation and actual contribution to board discussions (Forbes & Milliken, 1999; Wageman, 1995) will be stronger predictors of board effectiveness in individualistic than in collectivistic societies, since effort norms enforce the commitment that otherwise would be difficult to reach.

Consistent with previous research, hierarchy prevails over active participation in decision-making in high power distant cultures (Geletkanycz, 1997); cognitive conflicts are more likely to be suppressed than encouraged in such cultures. Hence, when conflicts arise, they lower hierarchical and cognitive barriers among board members (e.g., among outsiders and insiders) and create conditions for boards being participative in firm's critical decisions. Under such circumstances, conflicts have a stronger potential to engage board members in valuable discussions, which otherwise might be missing. This is perhaps one of the reasons why boards in Latin countries have a tradition for being passive; especially concerning control tasks (e.g., Brunello, Graziano, & Parigi, 2003).

Furthermore, different leadership styles in dissimilar national contexts will shape group dynamics in that they may stimulate or inhibit effective board processes. Hence, in high collectivist-low power distance cultures (like the ones characterizing Scandinavia), leadership style will encourage board participation, and processes will tend to be naturally more effective. As a consequence, however, it may lead to a “ceiling effect” in Scandinavian boards, since relatively less variance is achievable in a good process context. In sum, previous arguments point at the importance of developing effective board processes especially in those settings where autocratic leadership and low participative business styles prevail. In such settings, the greater variance in processes across boards will determine a higher predictive potential of the board processes themselves on board task performance. Thus, we hypothesize the following:

*Hypothesis 6*: Effort norms, cognitive conflicts and the use of knowledge and skills will have a stronger positive impact on both control and advisory task performance in Latin countries *vis-à-vis* Scandinavian countries.

### Methods

#### Sample and collection of data

The hypotheses were tested on data collected in Norway (representing Scandinavian countries) and Italy (representing Latin countries). The selection of two countries as proxies of cultures and institutions follows the tradition in cross-cultural organizational behaviour research. In this vein, issues related to cross-national data collection procedures, matching samples and construct equivalence often lead scholars to a choice of comparing two radically different countries (Tsui et al., 2007). Empirical literature on cross-country investigation has solid roots, and provided several examples of comparative research between two countries (e.g., Egri & Ralston, 2004; Lee, Bobko, Ashford, Zhen Xiong, & Xiaopeng, 2008; Lin, Peng, Yang, & Sun, 2009; Kirkman et al., 2009; Man & Lam, 2003). Cross-national studies typically consider countries featuring polarized characteristics in terms of the phenomenon under investigation, and consistently focus on culture and institutions as sources of variation. Based on previous examples, the selection of Norway and Italy has been inspired by their appropriateness in exploring the effects of national contexts on organizational processes within corporate boards. As argued theoretically, these two countries are markedly different along both the legal, the institutional, and the cultural dimensions. To provide further evidence supporting our arguments, we collected archival data from the World Competitiveness Report along several dimensions related to those traits. Following Wan & Hoskisson (2003), we computed two composite measures of both legal, and institutional and cultural dimensions to provide evidence of the differences between the two countries. Specifically, the legal variable includes: (i) the quality of the legal and regulatory framework and the judiciary system efficiency; (ii) the intellectual property protection and (iii) the competitive legislation (Wan & Hoskisson, 2003). The institutional and cultural variable includes: (i) political transparency; (ii) consumer price inflation and (iii) bribing and corruption (Wan & Hoskisson, 2003). While we could not use these variables in our regression analyses due to the presence of two countries in our dataset, the country-level scores support our theoretical assumptions about the significant differences between the two countries.

As Table 1 shows, the legal variable average score is 4.04 for Italy and 6.97 for Norway (out of a maximum of 10), and the gap is similar for the institutional and cultural variable, with a score of 2.70 for Italy and 5.58 for Norway (out of a maximum of 10). Table 1 also presents the differences on single items in order to show detailed variations on each dimension. This evidence provides additional support for the choice of the two countries, selected for this study.

**Table 1:** Legal and institutional/cultural differences in the two countries selected

	Legal and regulatory framework	Intellectual property protection	Competitive legislation	Legal variable (1–10)	Transparency	Consumer price inflation	Bribing and corruption	Institutional/cultural variable (1–10)
Italy	3.09	4.85	4.19	4.04	3.05	2.21	2.85	2.70
Norway	6.14	8	6.76	6.97	6.57	2.48	7.68	5.58
*δ*	−3.05	−3.15	−2.58	−2.92	−3.52	−0.27	−4.83	−2.87

*Source*: World Competitiveness Report; scores have been selected at the year the survey was issued.

Data from Norway and Italy was collected within a short time-span (autumn 2003 in Norway, spring 2004 in Italy). The Norwegian sample consists of the publicly listed firms and the one thousand largest industrial firms based on total turnover in 2002, totalling 1140 firms. The Italian sample consists of the 2000 largest Italian industrial firms ranked by turnover in the same year, including all the 240 industrial firms listed in that year at the Italian Stock Exchange. We obtained 379 answers from Norway, with a response rate of 33 per cent, and 301 answers from Italy, with a response rate of 15 per cent, which is in line with previous research on board of directors (Cycyota & Harrison, 2006). Additionally, we collected archival data on non responding firms in order to check for non-respondent bias. Data on firm characteristics were gathered from public sources and company annual reports. Specifically, we performed the non-parametric two independent samples test using the Kolmogorov-Smirnov procedure on firm size. We compared size in terms of annual turnover and number of employees of both respondent and non-respondent firms (Siegel & Castellan, 1988). The results of the tests provide evidence that respondents and non-respondents come from the same population in both sub-samples.

Following recommendations on matching samples in cross-national research (Tsui et al., 2007), we built a unique dataset considering firms of comparable size in both countries. We investigated only firms that in both samples had more than 50 employees, which represents a threshold for identifying medium and large firms (OECD Definition, 2005). The final dataset includes a total of 535 firms: 256 Norwegian firms (out of 379) and 279 Italian firms (out of 301). The two samples are comparable both in terms of number of firms included and in terms of average firm size. The average turnover for Norwegian firms is 1516 million euro, while it is 1977 million euro for Italian firms.

The survey data was based on responses from CEOs on behalf of the entire board. Since it is traditionally difficult to gain access to process data on boards of directors (e.g., Daily et al., 2003; Pettigrew, 1992), governance studies incorporating primary data are often based on a single respondent, typically the CEO (e.g., Pearce & Zahra, 1991; Zahra, 1996; Zahra, Neubaum, & Huse, 2000). In line with previous studies, we consider the CEO as the best possible key informant because he/she is knowledgeable about the issues investigated in our study, while at the same time he/she is also in a better position than other board members to report on them. Board members are part of a group which meets episodically, which suggests potential downsides of having multiple respondents. According to some scholars, having multiple reports in some specific circumstances can enhance the risk of constructing averaged measures which reflect divergence across reports, rather than representing the constructs being investigated (Kumar, Stern, & Anderson, 1993).

The dataset has been built through a survey instrument designed around established scales from the group effectiveness literature. In order to increase reliability of the data, we applied a number of procedural steps in the instrument development and data collection phase. First, we protected the respondents' anonymity by assuring confidentiality of their responses in the cover letter that accompanied the survey. Second, we invested considerable time and effort in improving the scale items and reducing item ambiguity. All survey questions are short, specific and use simple words to avoid ambiguous and vague formulations (Dillman, 2000). In some cases, following suggestions by Forbes and Milliken (1999), we adapted our items to the specific context of boards as decision-making groups. Related to this, in order to enhance the construct validity of the survey measures, we conducted a pre-test (Fowler, 1993: 102) and interviewed board members participating in the pilot study in Norway to assist us in the fine-tuning of the questionnaire, and particularly in identifying potentially misleading items (Carpenter & Westphal, 2001). Each individual was then asked to identify questions that were unclear, ambiguous or difficult to answer. Moreover, using inputs from the pilot interviews, we carefully worded questions to minimize the likelihood of a social desirability bias. Based on the survey instrument, developed in mid-2003, we sent out a traditional mailing to all the firms in our initial populations. In order to increase response rates, we had two rounds of reminders and we re-sent questionnaires to non-responding CEOs after two months. The collection procedure was identical in the two countries.

#### Measures

##### Dependent variables

Most of the dependent and independent variables are based on multiple-item constructs measured through a five point Likert-type scale. *Control task performance* was measured through five items. CEOs were asked to assess the extent to which the board: (i) controls that activities are well organized; (ii) establishes plans and budgets for the firms' operations; (iii) establishes guidelines for the operations of the firms; (iv) keeps itself informed about the financial position of the firm and (v) oversees that the operations are properly controlled (Huse 1993; Zona & Zattoni 2007). The variable control task performance was computed as a mean of these items, and the Cronbach *α* for this variable is 0.83.

*Advisory task performance* was also measured using five items which represent the different aspects boards of directors are supposed to contribute to. Accordingly, we used five statements about the degree to which the board provides advice on: (i) Management issues (e.g., organizational structure or company strategy); (ii) financial issues (e.g., leverage or relationships with banks and other financial institutions); (iii) technical issues (e.g., new technologies or products); (iv) market issues (e.g., entry in new industries or consumer behavior) and (v) legal issues and taxation (Minichilli & Hansen, 2007; van Ees, van der Laan, & Postma, 2008). The variable advisory task performance was computed as a mean of these items, and the Cronbach *α* for this variable is 0.78.

To assess firm financial performance we used *Return on Assets* (ROA), defined as the net operating income before extraordinary items divided by total assets. ROA is a well understood and common measure used in several studies on the impact of boards and top management teams on firm performance, and is particularly appropriate for manufacturing firms (e.g., Cannella & Shen, 2001; Carpenter, 2002; Finkelstein & D'Aveni, 1994; Geletkanycz & Hambrick, 1997; Henderson, Miller, & Hambrick, 2006). However, since the impact of executives on corporate level outcomes can be detected only within an appropriate performance window, we relied on a lagged value of ROA(*t* + 1), considering the year following the one in which the survey was administered (Shen, 2003; Westphal, 1999).

##### Independent variables

The independent variables included in the study are effort norms, cognitive conflicts and use of knowledge and skills. For *effort norms* we used three-items construct based on Forbes and Milliken (1999: 494). Effort norms was measured by asking the CEO the extent to which board members: (i) Carefully scrutinize information provided by management before the meetings; (ii) find their own information in relation to firm-specific issues and (iii) actively participate with critical questions during meetings. The above items are related to Wageman (1995); however, while Wageman's (1995) operationalization of effort norms seems to capture the level of individual effort expected from each of the members of the group, Forbes and Milliken's (1999) seems to capture more the actual behaviors of board members in terms of the effort they place on doing their tasks. Hence, our choice to rely on the latter items is in line with our purpose to focus on actual board behavior. The variable was computed as a mean of the three items, and the Cronbach *α* is 0.70. The *cognitive conflicts* construct has been developed for the purposes of this study based on Jehn's (1995) operationalization of conflicts, and relying on Forbes and Milliken's (1999) arguments to adapt such measure to the reality of corporate boards. Hence, cognitive conflicts was measured by asking the CEOs to evaluate the extent to which conflicts and disagreements emerged in the boardroom on: (i) Decisions to be taken during the board meetings; (ii) how to define what is the best for the firm; (iii) decision processes and (iv) firm's owners and stakeholders' interests. The Cronbach *α* for this variable is 0.86. Measurement for the *use of knowledge and skills* relies on what was first elucidated by Hackman & Morris (1975), and has been adapted to the board context following Forbes and Milliken (1999: 496). As such, it was measured by asking the CEO about the extent to which (i) board members know each others competences well; (ii) the division of work in this board is a good match between board members' knowledge/competencies and the character of the work and (iii) when an issue is discussed, the most knowledgeable board members use their knowledge. The Cronbach *α* for this variable is 0.76.

We used a dummy variable to capture country effects (Italy = 1, Norway = 0) and to assess the influence of the national context on board task performance, as well as its moderating effect on the board processes and task performance relationship. Similar to what the majority of cross-cultural studies do (Tsui et al., 2007), we consider country as a proxy for culture and institutions. To this end, all the interaction variables (country*effort norms; country*cognitive conflicts; country*use of knowledge and skills) were computed as a product of the originating process variables with the country dummy. To avoid collinearity, interaction variables have been built as the product of the country dummy by the mean-centered originating variable.

##### Control variables

We adopted both firm- and board-related control variables in our analyses. At the firm-level, we controlled for firm size, listing and age. Firm size was measured as firm turnover, and a logarithmic transformation allowed adjustment for skewness. We introduced a dummy variable to control for public listing (1 = listed company). Our firm age variable was a logarithmic transformation of the age the firm.

At the board-level, we controlled for CEO tenure and the traditional board demographic variables. CEO tenure may have an influence on board task performance across life cycle evolution (Shen, 2003). The CEO tenure was computed as the number of years in office the CEO served in the firm. The traditional board demographic variables included are the four “usual suspects”, i.e., board size, non-executive ratio, CEO duality and director shareholding (Finkelstein & Mooney, 2003). Board size was measured as the total number of directors (Zahra, Neubaum, & Huse, 2000). The non-executive ratio was measured as the percentage of non-executive directors over the total number of directors (Mallette & Fowler, 1992). The variable CEO duality was coded 1 if the CEO was also the chair of the board, and 0 otherwise (Finkelstein & D'Aveni, 1994). Director shareholding was measured as the ratio of director shareholding to total shareholding, and it included shareholding by inside directors (Kosnik, 1987; Zahra et al., 2000).

##### Validity of measures

In order to deal with potential common method bias (Doty & Glick, 1998), we performed some of the statistical remedies suggested by Podsakoff et al. (2003). First, we used the Harman's one factor test. The exploratory factor analysis of the items measuring board control and advisory task performance, as well as items underlying the process constructs (effort norms, cognitive conflicts, use of knowledge and skills), exhibited more than one factor with eigenvalues higher than 1.0, suggesting that the majority of the variance between the variables cannot be accounted for by one general factor (common method variance). Second, we used the partial correlation procedure to control for the effects of method variance (Lindell & Whitney, 2001). The results suggest that common method bias does not appear to be a problem in our data.

Additionally, all perceptual measures were evaluated in terms of reliability and validity. The psychometric properties of the multi-items constructs (effort norms, cognitive conflicts, use of skills and knowledge, control tasks and advisory tasks) were assessed simultaneously in one confirmatory factor analysis (CFA) using MPLUS Version 5.21. The CFA results showed a good model fit (CFI = 0.921; RMSEA = 0.062 with a 90 per cent confidence interval ranging between 0.056 and 0.069). We assessed reliability by calculating a composite reliability for each construct (Fornell & Larcker, 1981). Along with the reliability calculations, we also examined the parameter estimates and their associated t-values as well as the average variances extracted (Anderson & Gerbing, 1988). The reliabilities of the scales range from 0.91 to 0.97, the factor loadings range from 0.47 to 0.91 (*p* < 0.05), and the average variance extracted range from 76 to 90 per cent (see Table 2). The items were also found to be reliable and valid when evaluated based on each item's error variance, modification index and residual co-variation. Table 2 presents the results of the measurement assessment and reports the average variances extracted, construct reliabilities and factor loadings for all independent and dependent constructs used in our analyses.

**Table 2:** Summary statistics of the confirmatory factor analysis

	Average variance extracted (%)	Composite reliability	Range of loadings
1. Effort norms	78	0.91	0.47 to 0.85
2. Cognitive conflicts	90	0.97	0.65 to 0.91
3. Use of knowledge and skills	87	0.95	0.67 to 0.78
4. Control task performance	86	0.97	0.67 to 0.73
5. Advisory task performance	76	0.94	0.49 to 0.76

We established discriminant validity by two independent methods. First, we calculated the shared variance between each pair of constructs and verified that it was lower than the variances extracted for the involved constructs (Fornell & Larcker, 1981). The shared variances between pairs of all possible scale combinations indicated that the average variances extracted were higher than the associated shared variances in all cases. Second, we examined all possible pairs of constructs, as suggested by Bagozzi and Philips (1982), in a series of two-factor CFA models. The pairwise analysis tests showed that CFA models representing two factors fitted the data significantly better than one factor models (i.e., Δ *χ*^2^_(1)_ > 3.84 was exceeded in all cases). In addition, we tested for the invariability of the constructs and their measurement across the two national contexts. A multi-group analysis was conducted to validate the factorial structure of the proposed constructs and test whether the regression coefficients were invariant across the two populations (Williams, Edwards, & Vandenberg, 2003). We tested for equality with respect to the measurement model (invariance of factor loadings, factor variance and error terms) including all multi-items constructs across the two populations. The imposition of equality constraints on all freely estimated parameters led to multi-group comparison models with a good fit to the data. Furthermore, the results of the Lagrange-Multiplier test for incremental increase in *χ*^2^ when releasing equality constraints were all greater than 0.05, thereby indicating that the hypothesized equality of the specified factor loadings and factor variances held across the two samples. Hence, we found support for the validity of our constructs across the two subsamples.

### Results

Table 3 shows means, standard deviations and bivariate correlation coefficients for the variables used in the regression analyses.

**Table 3:** Correlation analysis

	1	2	3	4	5	6	7	8	9	10	11	12	13	14	15
1. Firm size	1														
2. Listing	0.14^*^^*^	1													
3. Firm age	−0.16^*^^*^	0.06	1												
4. CEO tenure	0.08	0.02	0.17^*^^*^	1											
5. Country (ITA = 1, NOR = 0)	0.22^*^^*^	0.07	−0.08	0.44^*^^*^	1										
6. CEO duality	0.03	0.05	0.02	0.42^*^^*^	0.34^*^^*^	1									
7. Board size	0.12^*^	0.25^*^^*^	0.11^*^	0.08	0.16^*^^*^	0.13^*^^*^	1								
8. Non-executives ratio	−0.06	0.16^*^^*^	0.10^*^	−0.32^*^^*^	−0.60^*^^*^	−0.27^*^^*^	0.24^*^^*^	1							
9. Director ownership	−0.06	−0.10	0.05	0.29^*^^*^	0.27^*^^*^	0.14^*^^*^	−0.21^*^^*^	−0.37^*^^*^	1						
10. Effort norms	−0.02	0.13^*^^*^	0.05	0.00	−0.09	−0.05	−0.13^*^^*^	0.07	0.03	1					
11. Cognitive conflicts	−0.03	−0.06	−0.04	−0.15^*^^*^	−0.21^*^^*^	−0.15^*^	0.00	0.12^*^^*^	0.06	0.20^*^^*^	1				
12. Use of knowledge and skills	−0.02	0.07	0.05	0.08	0.16^*^^*^	0.13^*^^*^	0.01	−0.17^*^^*^	0.15^*^^*^	0.19^*^^*^	−0.23^*^^*^	1			
13. Advisory tasks	0.01	0.11^*^	0.01	0.03	−0.04	0.05	−0.13^*^^*^	−0.06	0.12^*^	0.40^*^^*^	0.01	0.34^*^^*^			
14. Control tasks	−0.04	0.13^*^^*^	0.16^*^^*^	−0.12^*^	−0.42^*^^*^	−0.15^*^^*^	−0.03	0.23^*^^*^	−0.12^*^	0.36^*^^*^	0.01	0.26^*^^*^	0.52^*^^*^	1	
15. Roa (*t* + 1)	0.01	0.03	0.02	0.08	0.06	0.05	−0.01	−0.06	0.06	0.07	−0.09	0.19^*^^*^	0.22^*^^*^	0.18^*^^*^	1
Mean	11.80	0.20	3.59	11.76	0.49	0.19	6.62	0.67	27.26	3.07	1.90	3.88	3.15	3.75	6.06
*SD*	2.20	0.40	0.92	11.36	0.50	0.36	3.00	0.34	37.18	0.81	0.77	0.68	0.84	0.85	8.81

Pearson's product-moment correlation coefficients. 2-tailed: ^*^< 0.05; ^*^^*^< 0.01, *N* = 414.

Table 3 shows acceptable levels of correlations among the predictors and the dependent variables (board performance in control and advisory tasks). Based on this preliminary analysis, we conducted VIF analyses after each regression to test for multicollinearity. VIF values range from 1 to 3, indicating that multicollinearity is not a problem in our study (Neter, Kutner, Nachtsheim, & Wasserman, 1996).

The hypotheses were tested through hierarchical multiple regression analyses. Before running the analyses we examined potential problems in the distribution of variables with respect to assumptions of hierarchical regression analysis. We performed hierarchical regressions for the board tasks presented in the theory section. Each set of regressions was entered in different steps. The sets of regressions presented in Tables 4 and 5 have been performed in five different models: Model I includes control variables only; model II includes board demographics as board-related controls; model III considers the main effects of our predictors on board task performance; model IV includes the country effect and model V accounts for the three interactions among board processes and the country variable.

**Table 4:** Regression analyses for control task performance

Standardized *β* coefficients *N* = 415	Model I	Model II	Model III	Model IV	Model V
Controls					
Firm size	−0.07†	−0.06	−0.05	0.02	0.04
Listing	0.14^*^^*^	0.14^*^^*^	0.07	0.10^*^	0.10^*^
Firm age	0.20^*^^*^^*^	0.18^*^^*^^*^	0.15^*^^*^^*^	0.07†	0.07†
CEO tenure	−0.15^*^^*^^*^	−0.04	−0.05	0.06	0.04
Board demography					
CEO duality		−0.08	−0.08†	−0.05	−0.04
Board size		−0.12^*^	−0.06	0.08†	0.10^*^
Non-executives ratio		0.16^*^^*^	0.17^*^^*^^*^	−0.11†	−0.13^*^
Director ownership		−0.07	−0.10^*^	−0.06	−0.06
Board processes					
Effort norms			0.29^*^^*^^*^	0.28^*^^*^^*^	0.14^*^
Cognitive conflicts			−0.02	−0.07†	−0.13^*^
Use of knowledge and skills			0.23^*^^*^^*^	0.24^*^^*^^*^	0.32^*^^*^^*^
Country effect					
Country (ITA = 1, Nor = 0)				−0.53^*^^*^^*^	−0.55^*^^*^^*^
Interactions					
Country (ITA) ^*^ effort norms					0.18^*^^*^
Country (ITA) ^*^ conflicts					0.07
Country (ITA) ^*^ use of knowledge					−0.09
*R*^2^	0.08	0.12	0.26	0.38	0.41
Adj *R*^2^	0.07	0.10	0.24	0.37	0.39
*F* change	8.6^*^^*^^*^	4.91^*^^*^^*^	27.2^*^^*^^*^	81.3^*^^*^^*^	5.2^*^^*^
*F* (sign) full model	7.8^*^^*^^*^	6.9^*^^*^^*^	13.4^*^^*^^*^	21.5^*^^*^^*^	18.7^*^^*^^*^

† = 0.10-level, ^*^ = 0.05-level, ^*^^*^ = 0.01-level, ^*^^*^^*^= 0.001-level.

The listwise deletion procedure determined a number of complete observations of *N* = 415.

*Note*: Some changes have been made to this table to correct layout and reporting of decimal places on 7 February 2011 after first publication online on 5 January 2011.

**Table 5:** Regression analyses for advisory task performance

Standardized *β* coefficients *N* = 419	Model I	Model II	Model III	Model IV	Model V
Controls					
Firm size	−0.01	0.01	0.02	0.04	0.06
Listing	0.11^*^	0.15^*^^*^	0.07	0.08†	0.08†
Firm age	0.01	0.01	−0.03	−0.05	−0.05
CEO tenure	0.02	0.01	0.00	0.03	0.01
Board demography					
CEO duality		−0.01	−0.01	−0.00	0.02
Board size		−0.15^*^^*^	−0.08†	−0.04	−0.01
Non-executives ratio		−0.00	0.01	−0.08	−0.12†
Director ownership		0.10†	0.06	0.07	0.07
Board processes					
Effort norms			0.33^*^^*^^*^	0.33^*^^*^^*^	0.13^*^
Cognitive conflicts			0.01	−0.01	−0.12^*^
Use of knowledge & skills			0.28^*^^*^^*^	0.28^*^^*^^*^	0.34^*^^*^^*^
Country effect					
Country (ITA = 1, Nor = 0)				−0.17^*^^*^	−0.20^*^^*^^*^
Interactions					
Country (ITA) ^*^ effort norms					0.27^*^^*^^*^
Country (ITA) ^*^ conflicts					0.16^*^^*^
Country (ITA) ^*^ use of knowledge					−0.05
*R*^2^	0.01	0.05	0.25	0.26	0.32
Adj. *R*^2^	0.01	0.03	0.23	0.24	0.29
*F* change	1.2	3.9^*^^*^	37.5^*^^*^^*^	6.8^*^^*^	10.4^*^^*^^*^
*F* (sign) full model	1.2	2.6^*^^*^	12.7^*^^*^^*^	12.3^*^^*^^*^	12.6^*^^*^^*^

† = 0.10-level, ^*^ = 0.05-level, ^*^^*^ = 0.01-level, ^*^^*^^*^ = 0.001-level.

The listwise deletion procedure determined a number of complete observations of *N* = 419.

*Note*: Some changes have been made to this table to correct layout and reporting of decimal places on 7 February 2011 after first publication online on 5 January 2011.

The first set of regressions was performed considering the control task performance as dependent variable. As evident from Table 4, all the models are significant and adjusted *R*^2^ range from 0.08 (model I) to 0.41 (model V). Further, the most significant *F*-changes are between model II and model III (27.2***) and model III and model IV (81.3***), indicating the relevance of board processes and the country variable to predict control task performance. Table 4 shows results in details.

The second set of regressions refers to the board advisory task performance. As Table 5 shows, all models are significant with the exception of model I including only controls. Adjusted *R*^2^ range from 0.01 (model I) to 0.32 (model V), and the most significant *F*-change is between model II and model III (37.5***), indicating the predictive potential of board processes on board task performance in advice. Further, the *R*^2^ change and the *F*-change statistics between model IV and model V are stronger than the previous set of regressions, indicating that interactions are more relevant for advisory tasks than for control. Detailed results are presented in Table 5.

The third set of regressions tests for the hypothesized positive effects of board effectiveness in performing its tasks on firm's financial outcomes. Table 6 reports results on the sets of tests we performed in two different steps 1. The first model includes control variables, and both control and advisory task performance measures. The second model includes board processes in addition to control variables, and board task performance in control and advice to assess whether results from the first step remain robust after including our main predictors.

**Table 6:** Regression analyses for firm performance

Standardized *β* coefficients	Dependent variable = ROA *t* + 1				
Model I			Model II				
Control variables				
Firm size	0.07	0.08	0.10†	0.11†
Listing	0.04	0.01	0.03	0.01
Firm age	−0.02	−0.02	−0.03	−0.03
CEO tenure	0.05	0.09	0.05	0.09
CEO duality	0.04	0.06	0.02	0.04
Board size	−0.06	−0.02	−0.07	−0.04
Non-executives ratio	0.02	0.01	0.02	0.01
Director ownership	0.03	0.06	−0.01	0.02
Processes				
Effort norms			−0.02	−0.04
Cognitive conflicts			−0.03	−0.06
Use of know and skills			0.14^*^	0.10
Board tasks				
Control tasks		0.15^*^		0.13†
Advisory tasks		0.13†		0.15†
*R*^2^	0.02	0.09	0.04	0.11
Adj. *R*^2^	0.00	0.06	0.00	0.07
*F*/*F* change	0.44	10.24^*^^*^^*^	1.89	6.02^*^^*^
*N*=	320	320	304	304

† = 0.10-level,^*^ = 0.05-level, ^*^^*^ = 0.01-level, ^*^^*^^*^ = 0.001-level.

*Note*: Some changes have been made to this table to correct layout and reporting of decimal places on 7 February 2011 after first publication online on 5 January 2011.

Overall, our results show that: (i) Board performance in both control and advisory tasks predict firm financial performance measured as *Return On Assets* in year *t* + 1; and (ii) the positive effects of board task performance (both control and advice) remain significant even after introducing board processes in the regression equations 2. Overall, these results provide evidence that there is a relationship between self-reported performance measures of board tasks and firm performance, and that this relationship is robust (Simons, Pelled, & Smith, 1999).

**Figure 1:**
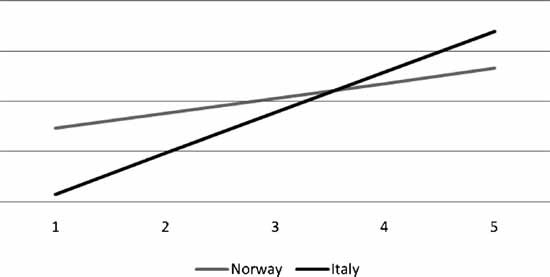
Board processes (effort norms) and control tasks in the two different countries

**Figure 2:**
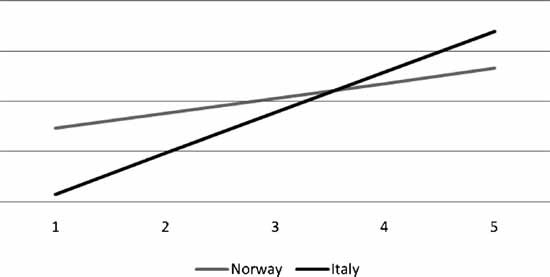
Board processes (effort norms) and advisory tasks in the two different countries

**Figure 3:**
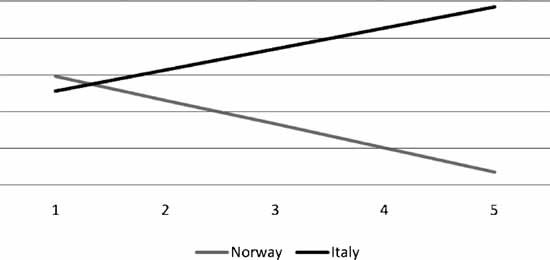
Board processes (cognitive conflicts) and advisory tasks in the two different countries

The different sets of regressions above provide support for hypotheses 1 and 3, respectively, on effort norms and the use of knowledge and skills, while they do not provide supporting evidence for Hypothesis 2. The results also provide support for Hypothesis 4 on the relationship between task performance and firm financial results. Furthermore, our results provide support for Hypothesis 5 on the higher board performance in both control and advisory tasks in Norway. With respect to Hypothesis 6, results show support for effort norms related to both board performance in control and advisory tasks, while cognitive conflicts show positive interaction with control task performance only. The use of knowledge and skills, however, shows no interactions at the country level with either control or advisory task performance. All the significant relationships are graphically presented in Figures 1–3 illustrating the trends of our process predictors with respect to board control (Figure 1) and advisory task (Figures 2 and 3) performance. Slopes in the figures have been plotted following guidance from Aguinis and Gottfredson (2010), and tests for slopes' significance have been realized using the Johnson–Neyman technique for probing interactions in linear models (Hayes and Matthes, 2009).

We performed an additional set of analyses in order to further validate our main results. Specifically, in order to account for potential perceptual biases of CEO respondents being also Chairs of their boards, we performed the same full set of regression analyses without considering responses of “dual” CEOs (i.e., CEOs being also board Chairs). While separate CEO and Chair positions may indicate CEOs as both “clients” of board advice and “targets” of board control, dual CEOs may be evaluating at least to some extent the quality of his or her own leadership of the board. Empirical analyses showed identical results for analyses excluding dual CEOs (the full analyses are not reported here). Results also show that dual CEOs are not positively biased in their ratings.

### Discussion

The main purpose of this study is to introduce a macro-level variable (country) to the boards and governance research on micro-level behavioral processes (Bamberger, 2008; Hambrick et al., 2008). We aim at understanding how board effectiveness is influenced both by micro-level board processes and macro-level institutional contexts simultaneously. We collected board data from two countries which substantially differ along both legal, and institutional and cultural dimensions in order to investigate how contexts moderate the relationship between board processes and task performance (La Porta et al., 1998). In this way, we offer empirical evidence on the co-existence of micro- and macro-level analyses in understanding board effectiveness within corporate governance structures (Lubatkin et al., 2005), and go beyond the mere acknowledgment of context as a potentially relevant variable (Bamberger, 2008).

#### Micro-level determinants of board task performance

A first set of results relates to the micro-level determinants of board task performance. Following recent calls to go beyond the formal structure of corporate boards (Daily et al., 2003; Hambrick et al., 2008), this study considers board effectiveness as the actual performance of board control and advisory tasks. We focus on micro-level board processes rather than demographics (Finkelstein & Mooney, 2003), and apply an integrated model of board effectiveness which considers three main board processes (Forbes & Milliken, 1999) as determinants of board task performance.

The results of our analyses support the validity of the applied model. First, consistent with our theoretical predictions, board demographics show limited impact on board task performance, indicating that they hardly predict board task performance or even firm performance (Daily et al., 2003; Hambrick et al., 2008). Some evidence emerges only with respect to non-executive ratio, illustrating how the presence of non-executives on boards is beneficial for control (Mallette & Fowler, 1992). However, this evidence applies only to basic models and changes direction of causality when we introduce interactions. Hence, the presence of non-executives seems to be only marginally relevant and even negative for advice, indicating how an unbalanced mix of directors toward outsiders favor board control yet at the price of lower inside knowledge of the firm (Mallette & Fowler, 1992).

Second, and most importantly, our findings provide support for board processes being much stronger predictors of board tasks than board's characteristics in terms of CEO duality, board size, board members' active shareholding or non-executive ratio (Finkelstein and Mooney, 2003). Specifically, effort norms show a consistent positive effect on both control and advisory task performance. This finding supports arguments pertaining to the benefits of board members doing their “homework” to understand firm's specificities and key strategic problems (Forbes & Milliken, 1999). Moreover, these findings are consistent with reviews of best practices to empower corporate boards (Lorsch & MacIver, 1989) and encourage open debate and enactment (Hambrick et al., 2008) in counteracting groupthink, thereby enhancing board effectiveness (Sonnenfeld, 2002). It also supports the importance of engaging board members in discussion, thereby avoiding “rubber stamping” attitudes toward managerial proposals (Lorsch & MacIver, 1989; Mace, 1971; Stiles & Taylor, 2001). Effort behaviors furthermore counteract habits of “pluralistic ignorance” in workgroups that potentially affects outside members in boards, who are often hesitant to express and share their concerns to others (Westphal & Bednar, 2005). Similar considerations apply to the use of knowledge and skills, as our results support the benefits of an active use and integration of board members' expertise and skills for group decisions. The use of knowledge and skills may prevent “process losses” and help interdependent decision-making groups, such as corporate boards, to build on each other's contributions (Forbes & Milliken, 1999).

The findings about cognitive conflicts indicate more complex patterns. While cognitive conflicts are not significant as a standalone effect, models including interactions show a negative association between conflicts and both control and advisory task performance. Although this evidence must be interpreted cautiously given potential perceptual biases related to measurement issues (Staw, 1975), it opens up for debate at both the micro- and the macro-level of analysis. At the micro-level, De Dreu and Weingart (2003) notice that, despite mainstream theory predictions support task-related conflicts as being positive for task effectiveness, a meta-analysis on the most recent empirical research questions this prediction. In terms of corporate boards, the customary reluctance toward open and candid discussion (Hambrick et al., 2008) can make conflicts an anguished experience for board members. The most important theoretical implications result from considering the macro-level of analysis, which suggests cognitive conflicts as being context-specifically relevant. This evidence reinforces our contribution to bring context into organizational research, and will be discussed in the following sections.

#### Macro-level (context) determinants of board task performance

A second set of results shows the impact of macro-level institutional context on board task performance. According to institutional theory, human and social behaviors are driven by the pervasive influence of institutions such as norms, rules, routines and historical patterns (DiMaggio & Powell, 1983; Scott, 2001). To test the hypothesized relationships we adopted a cross-national or cross-cultural lens (Gomez-Mejia & Wiseman, 2007) to examine how institutional settings and related institutional constraints shape the way boards perform their tasks. Our results provide evidence of the positive effect of the Scandinavian institutional context on board control and advisory task performance. This finding supports arguments of the relevance of legal (La Porta et al., 1998) and cultural (e.g., Hofstede, 1983) dissimilarities to board effectiveness at the micro-level. It further suggests that corporations are influenced not only by legal compliance with governance codes and statutes (Aguilera & Cuervo-Cazurra, 2004), but also by normative conformance with cultural and institutional norms and values on what represents effective governance (Hambrick et al., 2008). As such, this study provides a preliminary answer to theoretical calls for going beyond “context-free” or universalistic approaches to governance research (Aguilera et al., 2008).

#### The moderating effect of (macro-level) context on the relationship between board processes and board task performance

A third set of evidences reveals how macro- and micro-level determinants of board effectiveness interact to shape board task performance. As such, we address requests to explore how different institutional environments moderate the hypothesized relationships between sets of governance practices and organizational outcomes, such as effectiveness, efficiency or performance (Aguilera et al., 2008).

The strongest finding relates to effort norms, suggesting a stronger relevance of effort norms in contexts characterized by high power distance (Hofstede, 1984). The positive moderating effect on both control and advisory tasks (see Figures 1 and 2) further indicates the importance of a culture of commitment and preparation (Forbes & Milliken, 1999; Hambrick et al., 2008) in contexts where low participative decision-making culture prevails (Hofstede, 1984). Effort norms behaviors will encourage board member discussions rather than free-riding, and will make it more difficult for board members to hide behind “pluralistic ignorance” (Westphal & Bednar, 2005). Additional insights emerge when we consider the interaction effects of context on cognitive conflicts. While conflicts are found to negatively predict control and advisory tasks in the overall cross-country sample, they become positive in predicting advisory task performance of Italian corporate boards (see Figure 3). This evidence supports the assumed ambiguity of conflicts on group performance (De Dreu & Weingart, 2003), thus emphasizing the importance of context variables on organizational processes (Hambrick et al., 2008). We argue that in high power distance cultures, cognitive conflicts might help prevent “social distancing” among board members, and particularly between inside and outside directors (Westphal & Bednar, 2005). In this respect, the lack of evidence in relation to control may be explained by advisory tasks being more related to problem-solving, and hence requiring different perspectives and evaluation of different alternatives than cognitive conflicts may offer (Eisenhardt, 1989).

Finally, the use of knowledge and skills shows similar patterns in both contexts, with moderating effect being non-significant. Contrary to previous findings, our results show that using knowledge and skills in highly interdependent groups is predictive of group task performance regardless of the country context. As such, our findings point to the use of knowledge and skill as a more universalistic practice, which corporate boards should carefully follow. Overall, our results support theoretical predictions that effective board processes are likely to be stronger predictors of tasks in highly individualistic power distance cultures. Our findings may also relate to the greater variance in processes that individualistic cultures experience *vis-à-vis* collectivistic contexts. In other words, there might be a “ceiling effect” in high collectivist-low power distance cultures (like Scandinavia), since processes tend to be naturally higher in such cultures. Consequently, the relatively lesser variance in the good process context of Scandinavian boards will lower the predictive power of board processes on task performance.

#### Limitations and future directions

The paper is not without limitations, which may provide opportunities for future research. First, in line with the majority of previous cross-national studies (see Tsui et al., 2007), we investigated only two country contexts. Specifically, we considered Norway and Italy as examples of Scandinavian and Latin cultural and institutional environments which represent different cases in terms of corporate governance standards (La Porta et al., 1998). Including two legally and culturally distinct countries in our analyses assured adequate between-country variation on board effectiveness and internal processes. However, as Kirkman et al. (2009) and Tsui et al. (2007) noticed, including more countries may be appropriate to ascertain the generalizability of results, even in case of largely distinct countries, cultures and phenomena. Such an approach would allow researchers to examine within-country as well as between-country variance in board processes and task performance in a multilevel research design. It might also open up opportunities to investigate which specific factors at the country level serve as moderators of the board process-task performance relationships. Second, studies combining micro-level board process data with the macro-level determinants are rare, and more studies should be encouraged. As such, future studies should attempt to combine access to data with the need to go beyond the formal structure of boards in boards and governance research (Forbes & Milliken, 1999). Third, our study is cross-sectional due to difficulties in collecting cross-country data in relation to matching sample issues and construct equivalence (Tsui et al., 2007). Longitudinal studies may provide complementary and additional insights on how the evolution of the firm- and context- level variables influence the characteristics of governance practices. Future challenges include how processes can be measured across countries and time periods. Finally, this study is based on the perceptions of CEOs, and any generalization must take potential biases into account. Hence, it remains a challenge for future studies to explore the effects of perceptions by other board members and respondents (Hillman, Nicholson, & Shropshire, 2008). While these considerations impose some limitations on the interpretation of our results, they also offer valuable insights for future research in the area of international comparisons of board behaviors and practices, which is still considerably under-investigated.

### Conclusion

The primary objective of this study was to incorporate macro-level (country) explanations into the boards and governance research. To this end, we focused on board effectiveness in its tasks to investigate how macro-level determinants (context) influence micro-level relationships. Our analyses offer evidence of how both micro- and macro-level variables influence board task performance, and show how the macro-level country variable moderates relationships between board processes and task performance. As a result, the paper provides a preliminary answer to recent calls to bridge under-contextualized agency theory approaches and over-contextualized views of institutional theory (Aguilera et al., 2008; Aguilera & Jackson 2003; Hambrick et al., 2008), and more generally to incorporate macro-level variables in management research (Bamberger, 2008).

## Biographical Information

**Alessandro Minichilli** is Assistant Professor at Bocconi University in Milan, where he received his Ph.D. Alessandro has a background in corporate governance, with a focus on board characteristics and actual board behaviours. He is also interested in top management teams and executives' leadership characteristics. Based on this background, his recent work directs attention to family controlled firms, and lies in the intersection between corporate governance, TMTs and family business research. He teaches corporate governance and strategy in graduate courses.

## Biographical Information

**Alessandro Zattoni** is Professor of Management at Parthenope University in Naples and Professor of Strategic Management at the SDA Bocconi School of Management in Milan. His research interests focus on boards of directors, codes of good governance, stock incentive plans and ownership structures.

## Biographical Information

**Sabina Tacheva Nielsen** is a post-doctoral research fellow at the Department of International Economics and Management, Copenhagen Business School. She received her Ph.D. from University of St. Gallen, Switzerland. Her research interests include the composition, dynamics and effects of top management teams and boards, as well as diversity in multinational corporations.

## Biographical Information

**Morten Huse**, Professor dr. oecon, is affiliated with the Norwegian School of Management BI and Tor Vergata University in Rome. He was formerly the chairperson of the National Association of Corporate Directors (StyreAkademiet) in Norway, and he has been Director of International Themes at the Academy of Management in the USA. Morten Huse has published a large number of books and articles about behavioral perspectives on boards of directors.
